# *In utero* exposure to a mixture of phthalates, parabens, and other phenols and menstrual cycle characteristics in adolescents

**DOI:** 10.1016/j.ijheh.2025.114612

**Published:** 2025-06-29

**Authors:** Olivia P. Stoddard, Kimberly Berger, Brenda Eskenazi, Katherine Kogut, Nina T. Holland, Stephen Rauch, Kim G. Harley

**Affiliations:** a Division of Epidemiology, School of Public Health, University of California at Berkeley, Berkeley, CA, 94704, USA; b Sequoia Foundation, Berkeley, CA, 94710, USA; c Center for Environmental Research and Community Health (CERCH), School of Public Health, University of California at Berkeley, Berkeley, CA, 94704, USA

**Keywords:** Menstrual cycle, Endocrine disruption, Phthalates, Chemical mixtures, Reproductive effects, Bisphenol-A

## Abstract

Phthalates, parabens, and other phenols are present in consumer products humans use every day, including personal care products and food packaging. Exposure to these chemicals may have endocrine disrupting effects. The menstrual cycle is guided by the rise and fall of hormones, which may be disrupted by exposure to these chemicals. Urinary concentrations of metabolites of phthalates, parabens, and phenols in mothers during pregnancy and several menstrual cycle characteristics in their daughters at age 16 were examined in a predominantly Latino farmworker cohort. The association between the chemicals and each outcome was examined using logistic regression. Bayesian hierarchical modeling was used to model the chemical mixture’s associations with each outcome. All models were adjusted for poverty level during pregnancy, maternal pre-pregnancy BMI, and fastfood consumption at adolescent age 9. Results showed a positive association between mono (3-carboxypropyl) phthalate, propylparaben, and bisphenol-A and heavy menstrual flow. Exposure to 2,4-dichlorophenol was positively associated with short cycle length. Mono(3-carboxypropyl) phthalate and di (2-ethylhexyl) phthalate were positively associated with long cycle length. These results suggest *in utero* exposure to phthalates, parabens, and other phenols may be associated with abnormal menstrual cycle characteristics in adolescents.

## 1. Introduction/background

Endocrine disruptors are exogenous chemicals that block, mimic, or otherwise interfere with the body’s endogenous hormones, which are critical in maintaining physiological homeostasis (Endocrine Disruptors, n.d.). These chemicals disrupt reproductive hormones via the hypothalamic-pituitary-gonadal (HPG) axis and can have estrogenic, anti-estrogenic, androgenic, or anti-androgenic effects (Pan et al., 2024). Estrogen feedback occurs on the HPG axis and regulates events in the menstrual cycle such as follicular growth and ovulation (Koysombat et al., 2023). It is important to understand the effects of these chemicals on the menstrual cycle, as the menstrual cycle is increasingly recognized as a key indicator – sometimes referred to as a ‘vital sign’ – of overall health. Abnormal menstrual cycles can signal underlying issues with general ovarian function or reproductive health conditions such as polycystic ovary syndrome (PCOS), endometriosis, and infertility (Diaz et al., 2006).

Phthalates, parabens, and other phenols are classes of endocrine disrupting chemicals that are often found in plastics, personal care products, and cosmetics. Cosmetics and personal care products are the primary sources of exposure to low molecular weight (LMW) phthalates, which have been found in fragrances, soaps, lipsticks, and other cosmetics (Dodson et al., 2012; Wang et al., 2019). High molecular weight (HMW) phthalates are used in flexible plastics, food packaging, and vinyl materials (Wang et al., 2019). Parabens are commonly used in cosmetics and personal care products as preservatives (Dodson et al., 2012). Other phenols, including triclosan and benzophenone-3, are found in sunscreens, lotions, hairsprays, shampoos, and other personal care products (Han et al., 2016). Bisphenol-A (BPA) is another phenol commonly used in the manufacturing of plastics and resins (Cimmino et al., 2020). Exposure to these chemicals are widespread; in an average of the 2000–2010 National Health and Nutrition Examination Surveys (NHANES), phthalate metabolites were found in the urinary samples of 96 % of participants over 6 years of age (Zota et al., 2014). In the 2003–2004 NHANES, BPA was found in 93 % of participants (Calafat et al., 2008) and in the 2005–2006 NHANES, methylparaben was found in 99 % (Calafat et al., 2010).

Phthalates, parabens, and phenols have been associated with altered menstrual cycle characteristics and hormone function in prior studies. In humans, exposure to phenols and parabens have been associated with changes in progesterone, luteinizing hormone (LH), follicle stimulating hormone (FSH), and estradiol (Pollack et al., 2018). Urinary paraben concentrations were significantly associated with shortened menstrual cycle length in a study of healthy university students in Japan (Nishihama et al., 2016). Urinary concentrations of monocarboxyoctyl phthalate and BPA were associated with shorter luteal phases of the menstrual cycle in a sample of healthy women attempting pregnancy in North Carolina (Jukic et al., 2016). Animal and in vitro research corroborates epidemiological findings; phthalates have been found to disrupt the expression of estrogen-receptors in zebrafish (Chen et al., 2014; Xu et al., 2020) and parabens have shown estrogenic activity in rats and mice and in vitro (Witorsch and Thomas, 2010). A 2024 review of epidemiological and rodent-based studies found that paraben exposure may impact reproductive cyclicity and fertility (Pulcastro and Ziv-Gal, 2024). The prenatal period is particularly sensitive to endocrine disruption; developing biological systems are most sensitive to environmental exposures *in utero* and become less sensitive throughout childhood and into adulthood (National Scientific Council on the Developing Child, 2023). Exposure to phthalates, parabens, and phenols *in utero* may have impacts on reproductive development and health of the offspring, as reproductive organs are being rapidly developed and endocrine disrupting chemicals transfer to the fetus across the placenta (Mitro et al., 2015). During pregnancy, hormones regulate the development of the fetus’ sex organs and germ cells, which will become sperm or eggs, and play a key role in determining the future onset of puberty (US EPA, 2014). Ovary development begins in the first trimester, around week 5 of gestation. Key stages of ovary development, including germ cell proliferation and primordial follicle formation, occur between 5 and 20 weeks of gestation. Primordial follicle assembly begins around week 16 (Cox and Takov, 2025; Grive and Freiman, 2015). Prenatal exposure to parabens and other phenols have been associated with pubertal timing, including earlier age at menarche (Berger et al., 2018; Harley et al., 2019). Prenatal phthalate exposure has been associated with decreased serum hormone concentration and uterine volume in 16-year-old adolescents (Assens et al., 2024). These hormonal effects could potentially alter the normal menstrual cycle, which is guided by the rise and fall of estrogen, LH, FSH, and other hormones (Reed and Carr, 2000).

Despite growing evidence connecting endocrine-disrupting chemicals to altered hormone levels and menstrual health, no studies to our knowledge have assessed the longitudinal impact of *in utero* exposure to phthalates, parabens, and phenols on adolescent menstrual cycle characteristics. This study aims to fill that gap by investigating these associations and employing mixture modeling techniques to account for co-exposures and explore potential non-linear relationships. Specifically, we examined *in utero* urinary biomarker concentrations of metabolites of phthalates, parabens, and other phenols and their effects on menstrual cycle characteristics in adolescents using logistic regression and BHM. We hypothesized an association between *in utero* exposure to phthalates, parabens, and phenols and abnormal menstrual cycle characteristics in adolescents.

## 2. Methods

### 2.1. Study population

The Center for the Health Assessment of Mothers and Children of Salinas (CHAMACOS) is a cohort study that began in 1999 and included mothers and children living in the Salinas Valley of California, an agricultural area with a predominantly Latino immigrant population. The study continues to follow up participants and their children, with data being collected periodically since initial enrollment. Mothers were eligible for enrollment if they were age 18 or older, were English- or Spanish-speaking, qualified for Medicaid, and were planning to give birth at the county hospital. Mothers were interviewed twice during pregnancy to collect data including age, marital status, race/ethnicity, educational attainment, and household income (Eskenazi et al., 2010). Children were interviewed at multiple time points during childhood and adolescence, including at 16 years of age when they were asked about their menstrual cycle characteristics. Of the 606 children originally enrolled in this study, 274 were girls, of whom 171 completed the 16-year visit. We further excluded 1 girl who had not yet reached menarche by the 16-year visit, 1 who was missing menstrual data, 12 girls using hormonal contraception, 7 who did not have complete prenatal urinary biomarker data for phthalates, parabens, and other phenols, and 1 with outliers in the biomarker measurements for an analytical sample of n = 149 (Fig. S2).

### 2.2. Biomarker measurements

Maternal urine samples were collected in polypropylene urine cups at the end of the first trimester and the end of the second trimester of pregnancy, approximately 13 and 26 weeks gestation. The samples were then aliquoted into glass vials, stored at −80 °C, then shipped to the Centers for Disease Control and Prevention (CDC) in Atlanta, Georgia for analysis. Urinary biomarkers were measured via solid-phase extraction and isotope dilution high-performance liquid chromatography-–electrospray ionization–tandem mass spectrometry (Silva et al., 2007; Ye et al., 2005). Three LMW metabolites of phthalates were measured: monoethyl phthalate (MEP), a metabolite of diethyl phthalate; mono-n-butyl phthalate (MBP), a metabolite of di-n-butyl phthalate; and mono-isobutyl phthalate (MiPB), a metabolite of diisobutyl phthalate. Eight HMW phthalate metabolites were measured: monobenzyl phthalate (MBzP), a metabolite of benzyl butyl phthalate; mono-2-ethylhexyl phthalate (MEHP), mono-(2-ethyl-5-hydroxyhexyl) phthalate (MEHHP), mono-(2-ethyl-5-oxohexyl) phthalate (MEOHP), and mono-(2--ethyl-5-carboxypentyl) phthalate (MECPP), metabolites of di (2-ethylhexyl) phthalate (DEHP); monocarboxyoctyl phthalate (MCOP), a metabolite of di-isononyl phthalate; monocarboxy-isononly phthalate (MCNP), a metabolite of di-isodecyl phthalate; and mono (3-carboxypropyl) phthalate (MCPP), a metabolite of several HMW phthalates. Three parabens were measured: methylparaben, propylparaben, and butylparaben. Butylparaben was excluded from this study because of its low detection frequency in the cohort (<70 %). Five other phenols were measured: triclosan, benzophenone-3, 2,4-dichlorophenol (2,4-DCP), 2, 5-dichlorophenol (2,5-DCP), and bisphenol-A (BPA) (Berger et al., 2021). Where urinary biomarker concentrations were below the LOD, instrumental reading values were assigned when available. Otherwise, values were imputed by randomly selecting from the log-normal distribution using the maximum likelihood estimation (Lubin et al., 2004). Specific gravity was measured using a hand-held refractometer (National Instrument Company Inc., Baltimore, MD, USA) and the following formula was used to correct for variations in urine: (analyte concentration × [(1.024 – 1)/(sample specific gravity – 1)], where 1.024 is the reference urinary specific gravity for pregnant women (Harley et al., 2019; Mahalingaiah et al., 2008).

### 2.3. Menstrual cycle characteristics

The age 16 visit was chosen because all girls in the study had reached menarche by age 14, allowing at least 2 years post menarche for their cycles to stabilize. They were asked characterize their menstrual periods: whether their cycles were regular, defined as the number of days that pass between the start of one period and the start of the next period being about the same every time, give or take 4 days; cycle length (less than 21 days, 21–35 days, or more than 35 days); heaviness of menstrual flow (light, moderate, or heavy); severity of menstrual cramps (none; mild pain, defined as some loss of abilities to do things well; moderate pain, defined as in bed part of the day, some loss of school of work; or severe pain, defined as in bed all day for at least 1 day, not able to do anything), and the typical length of their period (1–2 days, 3–7 days, or more than 7 days) (Questionnaire S1).

### 2.4. Covariates

A directed acyclic graph was used to identify frequency of maternal fast food consumption, household poverty during the prenatal period, and maternal pre-pregnancy BMI as potential confounders (Fig. S1) (Greenland et al., 1999). Fast food consumption is a significant source of exposure to BPA and phthalates (Rudel et al., 2011; Schecter et al., 2013; Zota et al., 2016) and may affect the menstrual cycle via its impact on BMI (Bae et al., 2018; Tayebi et al., 2018). Because maternal fast food consumption was not observed in CHAMACOS, child fast food consumption at age 9 was adjusted for in analysis to block the backdoor pathway between the exposure and outcome via maternal fast food consumption. Child fast food consumption was measured via a questionnaire item about the number of times they ate fast food in the past week, with responses summarized as never, 1–3 times per week, or 4+ times per week. Poverty was categorized using household income at the first prenatal visit: at or below the federal poverty level, poverty level to 200 % above poverty level, and >200 % poverty level. Pre-pregnancy BMI was calculated using self-reported pre-pregnancy weight and height measured at the first prenatal visit. All models were adjusted for these variables.

### 2.5. Statistical analysis

Values used for each biomarker were calculated by averaging the measurements of two urinary samples collected during the mother’s pregnancy. All urinary biomarker values were log2-transformed and specific gravity adjusted to account for variability in urine from hydration status, muscle composition, and other factors. The molar masses of four metabolites of DEHP – MEHP, MEHHP, MEOHP, and MECPP – were summed into a ΣDEHP exposure variable since they are highly correlated and together account for approximately 60 % of DEHP exposure (Koch et al., 2005).

Cycle length, cycle regularity, period length, menstrual flow, and period pain were used as outcomes for the logistic regression and BHM models. Cycle length was dichotomized into two variables: normal (21–35 days) versus short (less than 21 days) and normal versus long (more than 35 days). Cycle regularity was measured as no variability month to month vs. variability month to month. Menstrual flow was divided into light or moderate vs. heavy. Period pain was dichotomized into no or mild pain and moderate to severe pain. Period length was dichotomized into normal (1–7 days) versus long (more than 7 days).

Logistic regression was used to estimate odds ratios between each individual chemical and outcome measure (single biomarker models) for comparison to the results of the Bayesian hierarchical model, and as the first stage of the two-stage Bayesian hierarchical modeling approach. BHM was used to generate log-odds in models that included all 15 biomarkers simultaneously, leveraging chemical class and functional knowledge to address co-pollutant confounding. We incorporated the log-odds from the logistic regression as our prior into the second stage of the BHM, which models the following equation:

β=Zπ+δ

Where Z is a matrix assigning each biomarker to its chemical class (LMW phthalate metabolites, HMW phthalate metabolites, parabens, or other phenols) using indicator variables with the expectation that biomarkers from the same chemical class would have similar effects on the outcomes, *π* is a vector of coefficients from the first-stage logistic regression, and *δ* is residual error assumed to be normally distributed with a mean of 0 and variance σ^2^. We set the standard deviation σ to equal 0.35, as researchers have previously done when applying BHM to binary outcomes (Berger et al., 2021; Bradshaw et al., 2013; Witte et al., 1994). This σ value reflects the prior belief that our odds ratios would range from 0.50 to 2.00. Analyses were completed in R version 4.3.1 (Vienna, Austria).

### 2.6. Ethical dimensions

The Institutional Review Board (IRB) of the University of California, Berkeley approved all study activities. Informed consent and parent permission were obtained from mothers at all study visits and informed written assent was obtained from daughters at age 16 years. The CDC IRB deferred approval to the IRB at the University of California, Berkeley (IRB approval number: 2010-03-949). The study authors are CITI-certified to conduct research involving human subjects.

## 3. Results

### 3.1. Study characteristics

Table 1 presents the characteristics of the cohort. Almost all maternal study participants self-identified as Latina (98.6 %). The majority of the cohort was at or below the poverty level before pregnancy (60.4 %) and at the 9 year follow-up visit (68.4 %). More than two-thirds of the mothers were overweight or obese at the time of pregnancy. Most of the mothers had an educational level of 12th grade or lower (80.5 %). About 59 % of the adolescent children consumed fast food one or more times per week and over half were overweight or obese. All but 5 adolescent participants had reached menarche by age 13. The remaining 5 reached menarche soon after their 14th birthday.

### 3.2. Chemical biomarker distributions and correlations

**Fig. 1.**
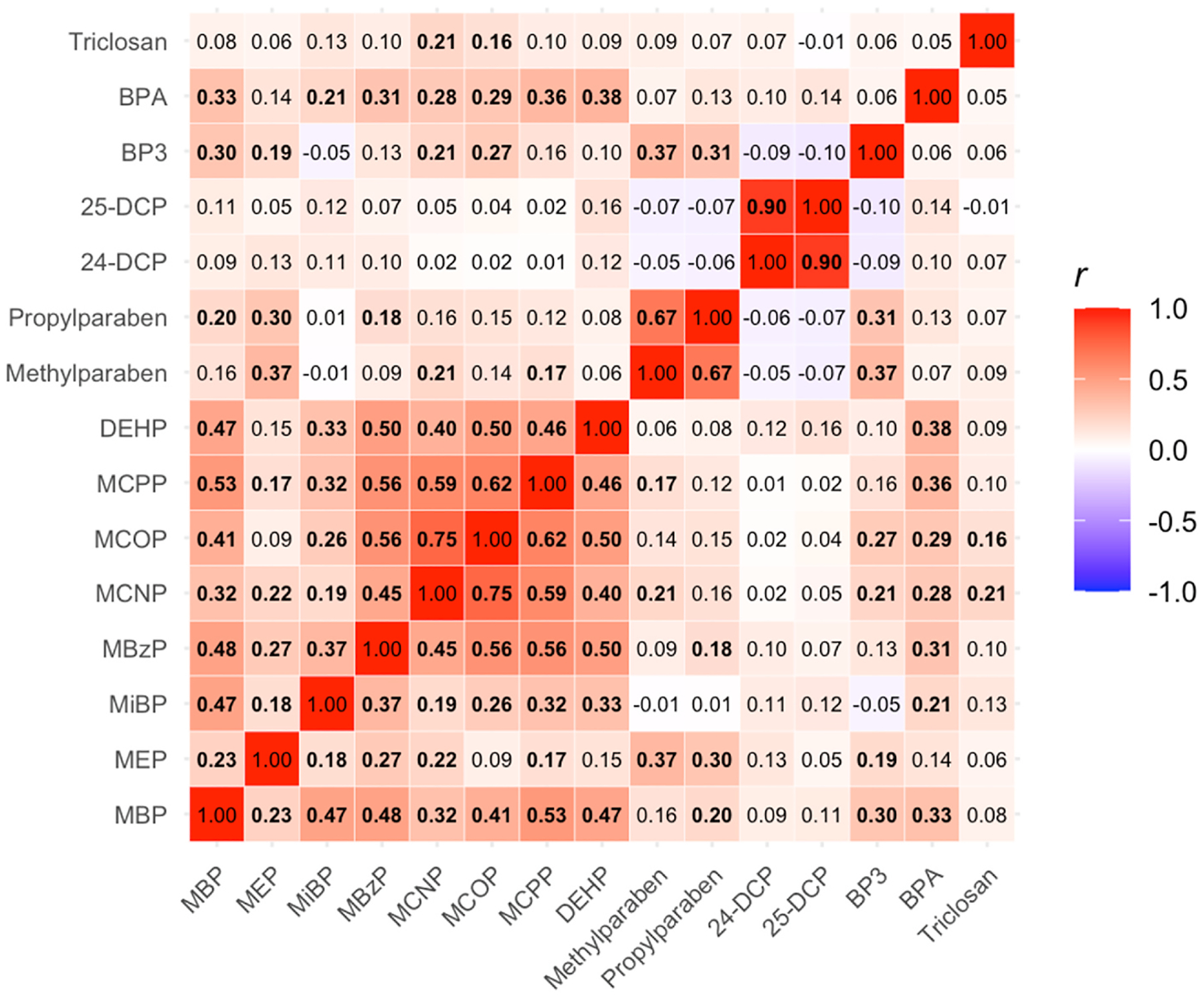
Pearson’s correlations of log2-transformed specific-gravity corrected phthalate metabolites, paraben, and other phenol urinary concentrations Abbreviations: MBP = mono-n-butyl phthalate metabolite; MEP = monoethyl phthalate metabolite; MiBP = mono-isobutyl phthalate metabolite; MBzP = monobenzyl phthalate metabolite; MCNP = monocarboxy-isononly phthalate metabolite; MCOP = monocarboxyoctyl phthalate metabolite; MCPP = mono (3-carboxypropyl) phthalate metabolite; DEHP = di (2-ethylhexyl) phthalate metabolite; 24-DCP = 2,4-dichlorophenol; 25-DCP = 2,5-dichlorophenol; BP3 = benzophenone-3; BPA = bisphenol-A. Statistically significant correlations are bolded.

Table 2 shows the distributions of the biomarker concentrations and the percent of urinary samples that had concentrations above the limit of detection. Limits of detection (LOD) ranged from 0.2 ng/mL to 2.3 ng/mL. All the biomarkers were detected in 80 % or more of the samples except for BPA and triclosan, which were on average present in approximately 78 % and 72 %, respectively. Biomarker measurements in CHAMACOS were comparable to pregnant women in NHANES of the same age (Table S2, Table S3). Fig. 1 shows Pearson’s correlations of log2-transformed specific-gravity corrected urinary concentrations of each biomarker. The biomarkers that are most correlated are 2,4-DCP and 2,5-DCP (*r* = 0.90, p < 0.0001), MCNP and MCOP (*r* = 0.75, p < 0.0001), and propylparaben and methylparaben (*r* = 0.67, p < 0.0001). Phthalates and parabens are more often correlated with other chemicals in their class compared to the other phenols. Pearson’s correlations between the two pregnancy samples were moderate for parabens and phenols and lowest for phthalate metabolites, ranging from 0.16 to 0.53 (Table S1).

### 3.3. Single biomarker and bayesian hierarchical models

Table 3 shows odds ratios from the single biomarker logistic regressions and the BHM.

#### 3.3.1. Heavy flow

Each doubling of urinary MCPP concentrations was associated with 55 % higher odds of heavy flow in the single biomarker model (OR: 1.55; 95 % CI: 1.05, 2.35) and 77 % higher odds in the BHM (OR: 1.77; 95 % CI: 1.10, 2.87). BPA was associated with 60 % higher odds of heavy flow in the single biomarker model (OR: 1.60; 95 % CI: 1.09, 2.41) but was not statistically significantly associated in the BHM. Propylparaben was associated with 21 % higher odds of heavy flow in the single biomarker model (OR: 1.21; 95 % CI: 1.03, 1.44) and 33 % higher odds of heavy flow in the BHM (OR: 1.33; 95 % CI: 1.07, 1.69).

#### 3.3.2. Short cycle length

MBP was associated with 38 % lower odds of short cycle length in the single biomarker model (OR: 0.62; 95 % CI: 0.40, 0.92) but was not statistically significantly associated in the BHM. Exposure to 2,4-DCP was associated with 43 % higher odds of short cycle length in the single biomarker model (OR: 1.43; 95 % CI: 1.13, 1.85) and 85 % higher odds in the BHM (OR: 1.85; 95 % CI: 1.18, 2.92).

#### 3.3.3. Long cycle length

MCPP was associated with 191 % higher odds of long cycle length in the single biomarker model (OR: 2.91; 95 % CI: 1.38, 7.39) but not statistically significantly associated in the BHM. ƩDEHP was associated with a 120 % increased odds of long cycle length in the single biomarker model (OR: 2.20; 95 % CI: 1.15, 4.87) but not statistically significantly associated in the BHM. Triclosan was associated with 23 % lower odds of long cycle length in the single biomarker model (OR: 0.77; 95 % CI: 0.57, 0.99) and 37 % lower odds of long cycle length in the BHM (OR: 0.63; 95 % CI: 0.43, 0.88).

#### 3.3.4. Cycle irregularity

Triclosan was associated with 15 % higher odds of cycle irregularity in the single biomarker model (OR: 1.15; 95 % CI: 1.01, 1.33) and 23 % higher odds of cycle irregularity in the BHM (OR: 1.23; 95 % CI: 1.05, 1.45).

None of the chemical biomarkers were statistically significantly associated with moderate/severe period pain or long period length.

## 4. Discussion

We found evidence that higher prenatal exposure to MCPP, propylparaben, and BPA were positively associated with heavy flow at adolescent age 16. MCPP and ƩDEHP were positively associated with long cycle length. We also observed a positive association between 2,4-DCP and short cycle length. MBP was negatively associated with short cycle length. Triclosan was negatively associated with long cycle length but positively associated with cycle irregularity.

Few studies have assessed the impact of *in utero* phthalates, parabens, and other phenols on menstrual cycle characteristics and, to our knowledge, no studies have examined this association in human adolescents using mixture methods. However, there are several relevant studies that investigate chemical exposures and reproductive function in humans and animals. Our results support findings that phthalates are associated with reproductive alterations, including longer cycle length, in mice (Patiño-García et al., 2018). Fletcher et al. observed an association between prenatal phthalate exposure and altered pro-apoptotic factors, disrupted steroidogenic enzymes, and reduced testosterone levels in F1 females, outcomes that are biologically linked with ovarian dysfunction. Such disruptions in ovarian function are plausible pathways leading to longer and heavier menstrual cycles in humans, supporting the biological relevance of our findings (2025). Our results also corroborate previous epidemiological findings that triclosan and 2, 4-DCP show endocrine disruption in a pubertal timing study in CHAMACOS (Harley et al., 2019). Pollack et al. used principal component analysis to examine the effects of urinary parabens, phenols, and UV filters on reproductive hormones (2018). Our results support their findings that phenols including 2,4-DCP were associated with decreased estradiol, as reduced estradiol levels are linked to shorter cycles (Mumford et al., 2012). Our results also corroborate their findings that paraben metabolites and BPA factor were associated with increased estradiol, which may be linked to cycle irregularity and menstrual flow (Attia et al., 2023). Our findings that MCPP is associated with heavy menstrual flow and both MCPP and ƩDEHP are associated with long cycle length are biologically supported by Assens et al., who observed prenatal exposure to phthalates was associated with altered hormone levels and reduced ovarian function in adolescent girls (2024). Disruption of ovarian development provides a mechanistic basis for the menstrual abnormalities associated with prenatal phthalate exposure in our study. While we saw associations between HMW phthalate metabolites and long cycle length, Jukic et al. saw an association between MCOP and shorter luteal phases of the menstrual cycle in women attempting pregnancy (2016). In a study of preconceptional women, high triclosan levels were associated with increased risks of abnormal menstruation, defined as less than 21 or more than 35 day cycle, while we observed a negative association between triclosan and long cycle length in the BHM (Zhu et al., 2019). However, these two studies did not measure prenatal chemical exposures.

This study has several notable methodological strengths, including a longitudinal study design and measures of multiple biomarkers at multiple timepoints. Biomarker data was collected during pregnancy, a critical period of the child’s reproductive system development. The use of two different mixture methods allows us to control for co-pollutant confounding and assess relationships within a more accurate representation of exposure to these chemicals. However, this study also has several limitations. Several of the odds ratios had wide confidence intervals, presumably from our small sample size and, in some cases, a very small number of participants with the outcome. There is also potential for residual confounding since we did not have information on all environmental chemical exposures that may impact menstrual cycle characteristics. The menstrual cycle may be additionally affected by exposure to endocrine disrupting chemicals after birth, during childhood. We did not have data on the adolescents’ childhood chemical exposure and thus cannot rule out the impact this exposure may have on their menstrual cycle characteristics. Phthalates and phenols metabolize quickly and the urinary measurements may not be indicative of average chemical exposure throughout the entire pregnancy. Correlations between measurements at approximately 13 and 26 weeks gestation were moderate for parabens and phenols but low for phthalates. Though we collected two measurements during pregnancy, additional measurements would have been preferred. Health effects may also occur in the first weeks of pregnancy, which our exposure timing did not capture. There is also potential selection bias from excluding participants who were on hormonal contraception. This may have excluded girls that had abnormal menstrual cycle characteristics, as contraceptives are sometimes prescribed to alleviate abnormal menstrual symptoms. However, the number of girls excluded for contraception use was minimal.

Finally, the self-report questionnaires on menstrual characteristics may be vulnerable to reporting bias. The adolescents were not asked to track their menstrual cycles and completed the questionnaire based on recall. Although the study participants were told their answers were confidential, adolescents may be embarrassed about their menstrual cycle due to social conditioning and alter their answers in a way that may bias the results towards the null. Additionally, period pain and heavy flow are subjective and many adolescents may not have adequate education on the menstrual cycle and may not track their cycle closely. Future research would benefit from using alternative data collection methods for menstrual cycle characteristics, such as the use of wearable technologies or cycle tracking on a mobile app to reduce these potential biases.

This study is one of the first of its kind and provides valuable information about the potential relationship between phthalates, parabens, and other phenols and menstrual cycle characteristics. The research landscape would greatly benefit from similar studies with larger sample sizes and minimally-biased data collection methods. Further research should investigate the effects of these chemicals on long cycle length, as this study found strong preliminary associations which may be indicative of anovulation. Future research should also consider the effects of chemical exposures on heavy flow, as this is a common symptom of endometriosis and associations between several prenatal chemical exposures and heavy flow were found in this study.

## Supplementary Material

1

## Figures and Tables

**Table 1:** Characteristics of study population: CHAMACOS cohort, Salinas, CA (N = 149).

		Mean (SD)+ or n (%)
Maternal age at delivery		26.8 (5.3)+
Maternal race/ethnicity	White	1 (0.7)
	Latina	147 (98.6)
	Other	1 (0.7)
Maternal pre-pregnancy poverty level	At or below poverty level	90 (60.4)
	Poverty to 200 % above poverty level	55 (36.9)
	>200 % above poverty level	4 (2.7)
Maternal education level	≤6th grade	69 (46.3)
	7–12th grade	51 (34.2)
	≥High school graduate	29 (19.5)
Household poverty at adolescent age 9	At or below poverty level	102 (68.4)
	Poverty to 200 % above poverty level	46 (30.9)
	>200 % above poverty level	1 (0.7)
Fast food consumption at adolescent age 9	<1 time per week	61 (41.2)
	1–3 times per week	83 (56.1)
	4+ times per week	4 (2.7)
Maternal pre-pregnancy BMI	Normal	47 (31.5)
	Overweight	45 (30.2)
	Obese	57 (38.3)
BMI at adolescent age 9	Normal	71 (48.0)
	Overweight	21 (14.2)
	Obese	56 (37.8)

**Table 2:** Distribution of specific-gravity adjusted biomarker urinary concentrations collected from mothers at two time points during pregnancy (N = 149).

Biomarker	% > LOD		Pregnancy Average (ng/mL)	Percentiles				
	1st trimester	2nd trimester	LOD	Geo. Mean	25 %	50 %	75 %	95 %
**LMW phthalate metabolites**								
MBP	97.3	99.3	0.4	30.6	18.3	29.0	47.6	124.1
MEP	98.6	98.7	0.6	231.5	105.5	219.1	520.1	1618.6
MiBP	94.6	96.6	0.2	3.8	2.0	4.1	6.8	16.6
**HMW phthalate metabolites**								
MBzP	96.6	98.0	0.3	9.0	4.8	9.4	17.8	41.6
MCNP	93.9	96.0	0.2	2.3	1.5	2.3	3.7	8.4
MCOP	95.3	96.0	0.2	3.8	2.2	3.6	6.1	11.6
MCPP	87.2	95.3	0.2	2.4	1.5	2.5	3.8	7.9
∑DEHP (nmol/mL)	NA	NA	NA	0.3	0.2	0.2	0.4	0.9
**Parabens**								
Methylparaben	96.6	95.3	1.0	142.8	67.3	207.6	445.6	727.2
Propylparaben	96.0	88.6	0.2	34.7	9.4	40.1	164.9	487.2
**Other phenols**								
2,4-Dichlorophenol	94.0	95.3	0.2	6.2	2.4	4.0	14.1	76.1
2,5-Dichlorophenol	97.3	94.6	0.2	77.7	17.5	74.1	496.8	1106.4
Benzophenone-3	96.0	95.3	0.4	34.8	5.9	21.6	323.9	1084.6
Bisphenol-A	74.5	81.9	0.2	1.5	0.9	1.3	2.4	6.1
Triclosan	69.1	75.2	2.3	27.7	7.0	20.7	181.4	727.8

Abbreviations: LOD = Limit of detection; LMW = low molecular weight; HMW = high molecular weight; MBP = mono-n-butyl phthalate metabolite; MEP = monoethyl phthalate metabolite; MiBP = mono-isobutyl phthalate metabolite; MBzP = monobenzyl phthalate metabolite; MCNP = monocarboxy-isononly phthalate metabolite; MCOP = monocarboxyoctyl phthalate metabolite; MCPP = mono(3-carboxypropyl) phthalate metabolite; DEHP = di(2-ethylhexyl) phthalate metabolite.

**Table 3:** Odds ratios and 95 % confidence/credible intervals for a doubling in each biomarker concentration and menstrual cycle characteristics in single-biomarker and Bayesian hierarchical models.

	Moderate/severe period pain		Heavy flow		Long period length	
	Single biomarker	Bayesian hierarchical	Single biomarker	Bayesian hierarchical	Single biomarker	Bayesian hierarchical
	OR (95 % CI)	OR (95 % CrI)	OR (95 % CI)	OR (95 % CrI)	OR (95 % CI)	OR (95 % CrI)
n	133		136		122	
**LMW phthalate metabolites**		1.00 (0.65, 1.55)		0.80 (0.51, 1.26)		1.02 (0.56, 1.84)
MBP	0.98 (0.73, 1.33)	1.07 (0.73, 1.59)	0.95 (0.67, 1.32)	0.77 (0.48, 1.21)	1.26 (0.63, 2.59)	1.06 (0.51, 2.19)
MEP	1.04 (0.85, 1.26)	1.07 (0.84, 1.34)	0.92 (0.73, 1.14)	0.89 (0.67, 1.19)	0.96 (0.60, 1.45)	1.01 (0.61, 1.62)
MiBP	0.89 (0.67, 1.17)	0.88 (0.64, 1.22)	0.81 (0.59, 1.11)	0.75 (0.51, 1.10)	1.31 (0.67, 2.66)	0.99 (0.52, 1.91)
**HMW phthalate metabolites**		0.98 (0.70, 1.36)		1.08 (0.76, 1.51)		1.22 (0.80, 1.93)
MBzP	0.80 (0.61, 1.04)	0.72 (0.50, 1.02)	1.01 (0.75, 1.37)	0.90 (0.60, 1.34)	1.15 (0.62, 2.23)	1.00 (0.56, 1.82)
MCNP	0.92 (0.66, 1.28)	0.87 (0.56, 1.33)	1.00 (0.69, 1.28)	0.75 (0.47, 1.20)	1.11 (0.50, 2.44)	1.01 (0.52, 1.96)
MCOP	0.89 (0.63, 1.25)	0.95 (0.61, 1.50)	1.12 (0.77, 1.64)	1.00 (0.61, 1.64)	2.13 (0.92, 5.59)	1.53 (0.79, 3.02)
MCPP	1.18 (0.84, 1.67)	1.47 (0.96, 2.25)	**1.55 (1.05, 2.35)**	**1.77 (1.10, 2.87)**	1.85 (0.84, 4.61)	1.42 (0.74, 2.76)
∑DEHP	0.94 (0.68, 1.30)	1.02 (0.68, 1.48)	1.17 (0.82, 1.69)	1.23 (0.80, 1.87)	1.35 (0.65, 2.88)	1.25 (0.66, 2.35)
**Parabens**		1.01 (0.61, 1.67)		1.10 (0.67, 1.84)		0.95 (0.54, 1.69)
MP	1.06 (0.87, 1.28)	1.08 (0.82, 1.42)	1.09 (0.87, 1.38)	0.92 (0.65, 1.29)	0.89 (0.56, 1.44)	0.94 (0.57, 1.55)
PP	0.98 (0.86, 1.11)	0.96 (0.80, 1.14)	**1.21 (1.03, 1.44)**	**1.33 (1.07, 1.69)**	0.96 (0.71, 1.33)	0.97 (0.66, 1.41)
**Other phenols**		1.04 (0.75, 1.43)		1.09 (0.79, 1.51)		0.88 (0.60, 1.29)
2,4-DCP	1.13 (0.94, 1.37)	1.19 (0.81, 1.74)	0.91 (0.94, 1.37)	0.98 (0.63, 1.50)	0.93 (0.55, 1.46)	0.86 (0.48, 1.52)
2,5-DCP	1.07 (0.94, 1.22)	1.03 (0.78, 1.37)	0.95 (0.82, 1.11)	0.99 (0.73, 1.33)	0.94 (0.67, 1.32)	0.91 (0.57, 1.43)
BP3	1.01 (0.90, 1.14)	1.01 (0.88, 1.17)	0.99 (0.86, 1.13)	0.95 (0.80, 1.13)	0.81 (0.54, 1.09)	0.75 (0.50, 1.07)
BPA	0.91 (0.65, 1.26)	0.93 (0.64, 1.33)	**1.60 (1.09, 2.41)**	1.53 (1.00, 2.34)	0.95 (0.42, 1.91)	0.77 (0.39, 1.50)
Triclosan	1.06 (0.94, 1.21)	1.08 (0.93, 1.24)	1.06 (0.92, 1.23)	1.11 (0.94, 1.32)	1.24 (0.90, 1.79)	1.20 (0.83, 1.76)
	Short cycle length		Long cycle length		Cycle irregularity	
	OR (95 % CI)	OR (95 % CrI)	OR (95 % CI)	OR (95 % CrI)	OR (95 % CI)	OR (95 % CrI)
n	95		73		118	
**LMW phthalate metabolites**		0.92 (0.58, 1.47)		0.97 (0.56, 1.68)		1.04 (0.67, 1.62)
MBP	**0.62 (0.40, 0.92)**	0.70 (0.42, 1.16)	1.09 (0.58, 2.02)	0.76 (0.37, 1.54)	1.12 (0.81, 1.55)	1.16 (0.75, 1.82)
MEP	1.10 (0.85, 1.43)	1.13 (0.78, 1.62)	1.33 (0.94, 1.94)	1.27 (0.78, 2.05)	0.92 (0.75, 1.12)	0.89 (0.69, 1.14)
MiBP	0.88 (0.63, 1.23)	1.00 (0.68, 1.48)	1.17 (0.72, 1.98)	0.94 (0.53, 1.68)	1.02 (0.77, 1.37)	1.07 (0.75, 1.54)
**HMW phthalate metabolites**		0.99 (0.70, 1.40)		1.48 (0.98, 2.27)		0.90 (0.64, 1.26)
MBzP	0.81 (0.58, 1.11)	0.90 (0.60, 1.36)	1.62 (0.98, 2.86)	1.52 (0.85, 2.75)	0.88 (0.67, 1.16)	0.87 (0.60, 1.26)
MCNP	0.75 (0.48, 1.15)	1.02 (0.62, 1.67)	1.18 (0.69, 2.04)	1.20 (0.65, 2.26)	0.86 (0.60, 1.22)	0.73 (0.46, 1.16)
MCOP	0.72 (0.45, 1.12)	1.08 (0.64, 1.81)	1.29 (0.72, 2.35)	1.11 (0.61, 2.09)	0.94 (0.66, 1.35)	0.94 (0.58, 1.51)
MCPP	0.72 (0.44, 1.16)	0.98 (0.58, 1.68)	**2.91 (1.38, 7.39)**	1.90 (0.98, 3.77)	0.85 (0.60, 1.21)	0.78 (0.49, 1.21)
∑DEHP	0.77 (0.51, 1.15)	0.97 (0.61, 1.54)	**2.20 (1.15, 4.87)**	1.82 (1.00, 3.41)	1.10 (0.80, 1.55)	1.26 (0.84, 1.91)
**Parabens**		1.00 (0.60, 1.68)		0.81 (0.46, 1.43)		1.11 (0.67, 1.83)
MP	1.00 (0.79, 1.28)	1.16 (0.80, 1.69)	0.85 (0.59, 1.25)	0.60 (0.35, 1.01)	1.14 (0.94, 1.41)	1.30 (0.97, 1.76)
PP	0.95 (0.82, 1.10)	0.88 (0.70, 1.10)	1.08 (0.87, 1.40)	1.09 (0.78, 1.53)	1.01 (0.89, 1.16)	0.93 (0.77, 1.13)
**Other phenols**		1.04 (0.74, 1.46)		0.87 (0.60, 1.26)		1.08 (0.78, 1.50)
2,4-DCP	**1.43 (1.13, 1.85)**	**1.85 (1.18, 2.92)**	1.00 (0.68, 1.44)	1.25 (0.70, 2.24)	1.03 (0.84, 1.26)	0.92 (0.60, 1.39)
2,5-DCP	1.16 (0.98, 1.39)	0.80 (0.58, 1.11)	0.88 (0.66, 1.14)	0.82 (0.53, 1.25)	1.05 (0.91, 1.21)	1.11 (0.83, 1.50)
BP3	0.92 (0.79, 1.07)	0.95 (0.78, 1.15)	1.02 (0.80, 1.29)	0.93 (0.67, 1.26)	1.09 (0.96, 1.24)	1.11 (0.95, 1.31)
BPA	0.92 (0.60, 1.39)	1.07 (0.67, 1.68)	1.19 (0.62, 2.27)	0.83 (0.44, 1.55)	0.97 (0.62, 2.27)	1.06 (0.72, 1.55)
Triclosan	0.85 (0.74, 1.02)	0.82 (0.67, 0.99)	**0.77 (0.57, 0.99)**	**0.63 (0.43, 0.88)**	**1.15 (1.01, 1.33)**	**1.23 (1.05, 1.45)**

Abbreviations: LMW = low molecular weight; HMW = high molecular weight; MBP = mono-n-butyl phthalate metabolite; MEP = monoethyl phthalate metabolite; MiBP = mono-isobutyl phthalate metabolite; MBzP = monobenzyl phthalate metabolite; MCNP = monocarboxy-isononly phthalate metabolite; MCOP = monocarboxyoctyl phthalate metabolite; MCPP = mono(3-carboxypropyl) phthalate metabolite; DEHP = di(2-ethylhexyl) phthalate metabolite; 2,4-DCP = 2,4-dichlorophenol; 2,5-DCP = 2,5-dichlorophenol; BP3 = benzophenone-3; BPA = bisphenol-A. Adjusted for poverty level during pregnancy, maternal pre-pregnancy BMI, and fast food consumption at adolescent age 9.

## Appendix A. Supplementary data

Supplementary data to this article can be found online at https://doi.org/10.1016/j.ijheh.2025.114612.
