# Supplementary material for: In utero exposure to a mixture of phthalates, parabens, and other phenols and menstrual cycle characteristics in adolescents
Source: Int J Hyg Environ Health. Author manuscript; Available in PMC 2026 May 10. (PMC13157871; doi:10.1016/j.ijheh.2025.114612)
Supplement: 1 [file NIHMS2169506-supplement-1.docx]

### Supplemental tables and figures

**Figure S1. Directed acyclic graph**

**
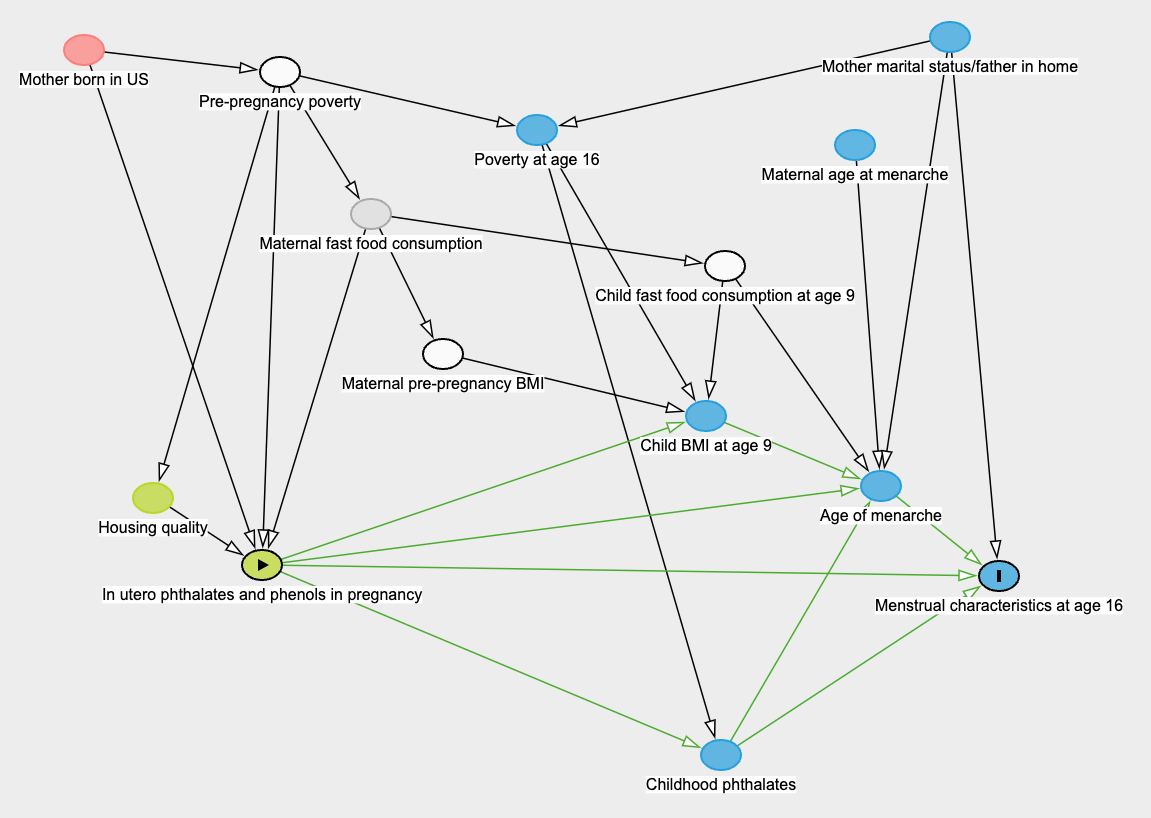
**

**Figure S2. Participant flow chart**

**
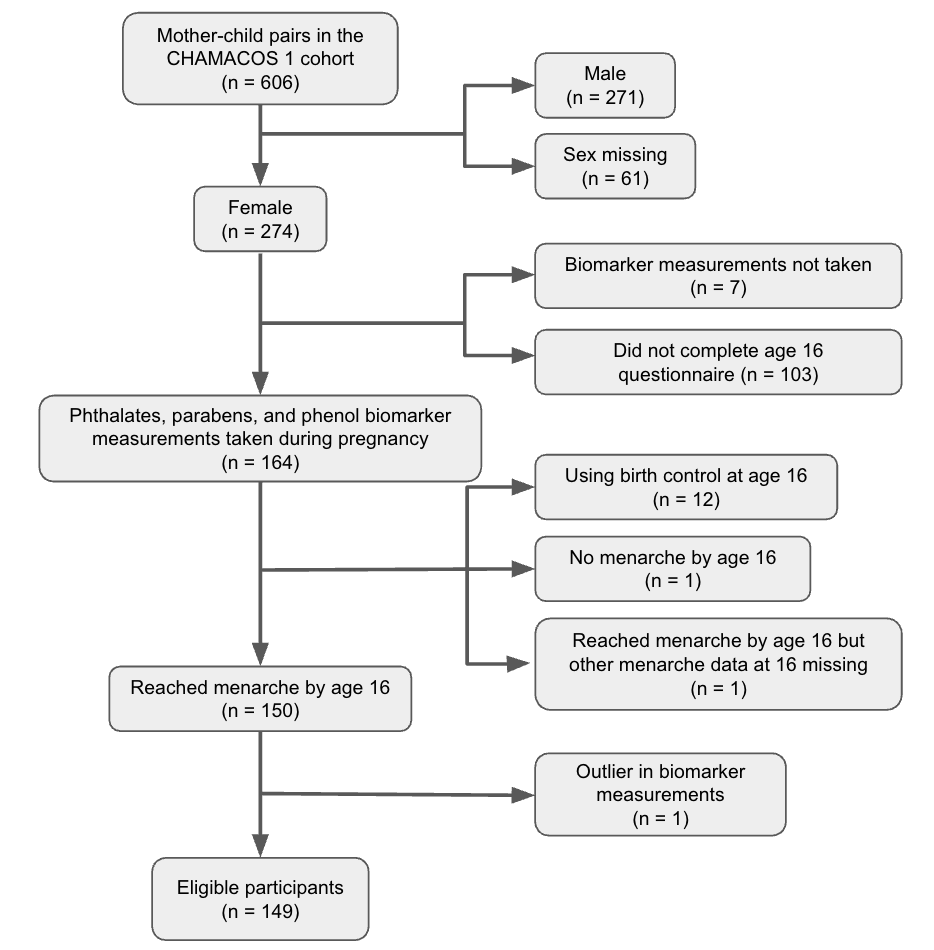
**

**Table S1. Pearson’s correlations of log2-transformed specific-gravity adjusted phthalate, paraben, and phenol measurements at 13 and 26 weeks gestation**

| **Biomarker** | **Correlation *r*** |
| --- | --- |
| **LMW phthalates** | |
| MBP | 0.17 |
| MEP | 0.32 |
| MiBP | 0.21 |
| **HMW phthalates** | |
| MBzP | 0.35 |
| MCNP | 0.24 |
| MCOP | 0.16 |
| MCPP | 0.21 |
| ƩDEHP | 0.20 |
| **Parabens** | |
| MP | 0.47 |
| PP | 0.46 |
| **Other phenols** | |
| 2,4-DCP | 0.46 |
| 2,5-DCP | 0.55 |
| BP3 | 0.53 |
| BPA | 0.18 |
| Triclosan | 0.40 |

**Table S2. Distribution of creatinine-adjusted paraben and phenol measurements in urine of CHAMACOS participants and pregnant women aged 18-43 years in NHANES (ng/mL)***

| **Biomarker** | **Cohort** | **Geo. Mean** | **25%** | **50%** | **75%** | **95%** |
| --- | --- | --- | --- | --- | --- | --- |
| **MP** | CHAMACOS | 126.60 | 51.79 | 151.66 | 332.16 | 685.40 |
|  | NHANES | 98.39 | 34.40 | 107.00 | 340.00 | 1140.00 |
| **PP** | CHAMACOS | 31.26 | 9.47 | 34.56 | 143.53 | 509.18 |
|  | NHANES | 17.26 | 5.30 | 19.80 | 86.50 | 407.80 |
| **BP3** | CHAMACOS | 30.30 | 5.12 | 21.41 | 247.78 | 891.38 |
|  | NHANES | 37.63 | 10.08 | 40.55 | 121.00 | 633.05 |
| **BPA** | CHAMACOS | 1.29 | 0.80 | 1.16 | 1.88 | 5.53 |
|  | NHANES | 2.56 | 1.43 | 3.00 | 4.43 | 12.4 |
| **Triclosan** | CHAMACOS | 23.30 | 5.84 | 16.96 | 148.09 | 559.67 |
|  | NHANES | 13.78 | 3.23 | 9.50 | 34.18 | 596.50 |

*CHAMACOS phenol and paraben measurements were measured from 1999-2001. Triclosan, BPA, and BP3 were measured in the 2003-2004 NHANES cycle; MP and PP were measured in the 2005-2006 cycle. 24-DCP and 25-DCP are not measured in NHANES.

**Table S3. Distribution of creatinine-adjusted phthalate metabolite measurements in urine of CHAMACOS participants and pregnant women aged 18-43 years in NHANES (ng/mL)***

| **Biomarker** | **Cohort** | **Geo. Mean** | **25%** | **50%** | **75%** | **95%** |
| --- | --- | --- | --- | --- | --- | --- |
| **MBP** | CHAMACOS | 25.64 | 15.23 | 23.09 | 45.30 | 82.94 |
|  | NHANES | 22.40 | 11.30 | 23.90 | 50.20 | 122.40 |
| **MEP** | CHAMACOS | 196.61 | 85.07 | 208.62 | 425.08 | 1380.60 |
|  | NHANES | 141.00 | 53.26 | 150.35 | 321.35 | 916.28 |
| **MiBP** | CHAMACOS | 3.11 | 1.80 | 3.26 | 5.51 | 11.84 |
|  | NHANES | 2.85 | 0.70 | 2.75 | 6.50 | 15.88 |
| **MBzP** | CHAMACOS | 7.57 | 4.53 | 7.93 | 13.81 | 26.04 |
|  | NHANES | 10.6 | 4.6 | 11.8 | 22.9 | 92.3 |
| **MCNP** | CHAMACOS | 1.98 | 1.31 | 1.81 | 2.83 | 6.72 |
|  | NHANES | 1.98 | 1.00 | 2.00 | 3.80 | 14.36 |
| **MCOP** | CHAMACOS | 3.31 | 2.12 | 3.41 | 5.17 | 9.32 |
|  | NHANES | 4.15 | 2.00 | 3.30 | 8.10 | 51.76 |
| **MCPP** | CHAMACOS | 2.02 | 1.43 | 2.18 | 2.99 | 6.46 |
|  | NHANES | 0.02 | 0.01 | 0.02 | 0.03 | 0.05 |
| **ƩDEHP (nmol/mL)** | CHAMACOS | 0.22 | 0.13 | 0-.20 | 0.34 | 0.76 |
|  | NHANES | 0.25 | 0.11 | 0.26 | 0.48 | 1.56 |

*CHAMACOS phthalate metabolites were measured from 1999-2001. MBP, MEP, and MBzP were measured in the 1999-2000 NHANES cycle. MiBP and MCPP were measured in the 2001-2002 NHANES cycle. ƩDEHP was measured in the 2003-2005 NHANES cycle. MCNP and MCOP were measured in the 2005-2006 NHANES cycle.

**Questionnaire S1. Menstrual cycle questionnaire administered to girl participants at age 16**

- The first questions are about your menstrual period. Have you had your first period yet?
  - No
  - Yes
- How many times have you had your period?
  - 1 time
  - 2 times
  - 3 times
  - 4+ times (or more than I can remember!)
- Some girls get a "regular" period, meaning that the number of days that pass between the start of one period and the start of the next period is about the same every time, give or take 4 days. Is your period regular?
  - No
  - Yes
  - Don’t know
- Your "cycle" is the number of days that pass between the start of one period and the start of the next period. About how many days is your cycle usually?
  - Less than 21 days
  - 21-45 days (individually listed)
  - More than 45 days
  - I don’t know
- In general, approximately how long is your cycle?
  - Less than 25 days (less than one month)
  - About 25-31 days (about one month)
  - More than 31 days (more than one month) I don't know
- Your period length is the number of days of bleeding during each period. In general, approximately how many days do you usually bleed during your period?
  - 1-2 days
  - 3-7 days
  - More than 7 days
  - Don't know or don't remember
- How would you describe the heaviness of menstrual flow during a typical period?
  - Light
  - Moderate
  - Heavy
- Some girls get "menstrual cramps" or feel pain during their period. How much pain, if any, do you feel during your period?
  - None
  - Mild pain (some loss of ability to do things well)
  - Moderate pain (in bed part of day, some loss of school or work)
  - Severe pain (in bed all day for at least 1 day, not able to do anything)
  - Don't know or don't remember
